# Open Partial Horizontal Laryngectomies for T3–T4 Laryngeal Cancer: Prognostic Impact of Anterior vs. Posterior Laryngeal Compartmentalization

**DOI:** 10.3390/cancers11030289

**Published:** 2019-03-01

**Authors:** Francesca Del Bon, Cesare Piazza, Davide Lancini, Alberto Paderno, Paolo Bosio, Stefano Taboni, Riccardo Morello, Nausica Montalto, Francesco Missale, Fabiola Incandela, Filippo Marchi, Marta Filauro, Alberto Deganello, Giorgio Peretti, Piero Nicolai

**Affiliations:** 1 Department of Otorhinolaryngology—Head and Neck Surgery, University of Brescia, Piazza Spedali Civili 1, 25123 Brescia, Italy; delbonfrancesca@gmail.com (F.D.B.), lancinidavide@gmail.com (D.L.); paolo.bosio92@gmail.com (P.B.); stefanotaboni@gmail.com (S.T.); dott.riccardomorello@gmail.com (R.M.); nausica.montalto@gmail.com (N.M.); adeganello@hotmail.com (A.D.); pieronicolai@virgilio.it (P.N.); 2 Department of Otorhinolaryngology, Maxillofacial and Thyroid Surgery, Fondazione IRCCS, National Cancer Institute of Milan, University of Milan, 20133 Milan, Italy; ceceplaza@libero.it (C.P.); Fabiola.Incandela@istitutotumori.mi.it (F.I.); 3 Department of Otorhinolaryngology, Head and Neck Surgery, University of Genoa—IRCCS Ospedale Policlinico San Martino, 16132 Genoa, Italy; missale.francesco@gmail.com (F.M.); filippomarchi@hotmail.it (F.M.); mfilauro@yahoo.com (M.F.); giorgioperetti18@gmail.com (G.P.)

**Keywords:** laryngeal cancer, open partial horizontal laryngectomy, conservative surgery, paraglottic space, prognosis

## Abstract

Open partial horizontal laryngectomies (OPHLs) are well-established and oncologically safe procedures for intermediate–advanced laryngeal cancers (LC). T–N categories are well-known prognosticators: herein we tested if “anterior” vs. “posterior” tumor location (as defined in respect to the paraglottic space divided according to a plane passing through the arytenoid vocal process, perpendicular to the ipsilateral thyroid lamina) may represent an additional prognostic factor. We analyzed a retrospective cohort of 85 T3–4a glottic LCs, treated by Type II or III OPHL (according to the European Laryngological Society classification) from 2005 to 2017 at two academic institutions. Five-year overall survival (OS), disease-specific survivals (DSS), and recurrence-free survivals (RFS) were compared according to tumor location and pT category. Anterior and posterior tumors were 43.5% and 56.5%, respectively, 78.8% of lesions were T3 and 21.2% were T4a. Five-year OS, DSS, and RFS for T3 were 74.1%, 80.5%, and 63.4%, respectively, and for T4a 71.8%, 71.8%, and 43%, respectively (*p* not significant). In relation to tumor location, the survival outcomes were 91%, 94.1%, and 72.6%, respectively, for anterior tumors, and 60.3%, 66.3%, and 49.1%, respectively, for posterior lesions (statistically significant differences). These data provide evidence that laryngeal compartmentalization is a valid prognosticator, even more powerful than the pT category.

## 1. Introduction

T3 and T4a laryngeal cancers (LC) represent a heterogeneous group of lesions amenable to partial and total laryngectomies, as well as non-surgical therapeutic strategies. Results of these different treatment options in terms of overall survival (OS), disease-specific survivals (DSS), and loco-regional control are deemed to be comparable. Nonetheless, functional outcomes in terms of quality of life, residual voice, and swallowing may be significantly different. However, prospective studies comparing different treatment strategies are still lacking and difficult to implement in the near future [1,2,3].

Historically, total laryngectomy (TL) had been considered the primary option for locally advanced LC. More recently, the Veterans Affairs and RTOG 91-11 studies demonstrated the efficacy of non-surgical options that led to the popularization of chemo-radiation (CRT) as an organ sparing treatment [4,5]. In general, the principal aim was to cure the cancer and preserve the larynx in order to provide an improvement of functional results. However, subsequent studies reported suboptimal long-term outcomes regarding airway patency and swallowing, highlighting the critical difference between “anatomic” and “functional” laryngeal preservation [6,7].

At the same time, conservative surgical techniques, comprising of both transoral laser microsurgery (TLM) and open-neck approaches (i.e., open partial horizontal laryngectomies (OPHLs)), have been developed and are widely used. Both treatments have shown sound and reproducible outcomes in different stages of LC [8,9,10,11,12,13,14]. However, patient selection plays a pivotal role in achieving optimal results and should take into consideration a number of patient- and tumor-related factors—this is especially true when considering OPHLs [15]. On one hand, patient comorbidities (pulmonary, neurological, cardiologic, and overall performance status) may contraindicate partial laryngeal surgery due to the postoperative risk of chronic subclinical aspiration and pneumonia. On the other hand, tumor extension should be carefully assessed—taking into account all major pathways of spread—in order to achieve a complete resection within clear surgical margins. As already demonstrated by data from independent reports, in carefully selected patients OPHLs represent solid and standardized surgical approaches, adaptable according to a number of tumor features, including its location [16,17,18,19,20]. In fact, Succo et al. [21] recently suggested tumor extension (distinguished in anterior vs. posterior involvement of the paraglottic space (PGS) according to an arbitrary plane passing at the level of the arytenoid vocal process and perpendicular to the ipsilateral thyroid lamina) as a major prognosticator in patients treated by OPHLs for T3–T4 LCs. The aim of the present study was to confirm this observation in an independent retrospective series of advanced glottic T3–T4a treated by OPHLs at two academic institutions.

## 2. Materials and Methods

A retrospective study was carried out in patients who underwent Type II or III OPHLs, according to the European Laryngological Society classification [16], for T3–T4a glottic squamous cell carcinoma (SCC) from January 2005 to July 2017 at the Departments of Otorhinolaryngology—Head and Neck Surgery, Universities of Brescia and Genoa (Brescia, Genoa, Italy). Purely supraglottic SCCs not approaching the vocal plane (and therefore to be treated by TLM or OPHL Type I) were excluded from the analysis. All data concerning comorbidities, preoperative staging, surgical results, tumor histology, adjuvant therapies, and follow-up were collected in a single dedicated database. Patients were re-staged using the 8th Edition of the TNM classification [22]. This was a purely retrospective study with no need to receive approval from an ethics committee, and no informed consent was signed.

Of 100 patients undergoing Type II or III OPHLs for laryngeal SCC, 85 were considered eligible for the study. Considering the excluded patients: 9 (2 pT1b and 7 pT2) received OPHL due to a suboptimal laryngeal exposure for TLM [23] and 6 had been previously treated by RT for laryngeal SCC.

The male to female ratio was 13:1 and the mean age at presentation was 60.8 years (range, 42–78). Thirty-seven (43.5%) patients had already been treated for LC by TLM and underwent OPHL for relapse (*N* = 34 or 40%) or second tumor (*N* = 3 or 3.5%).

**Figure 1:**
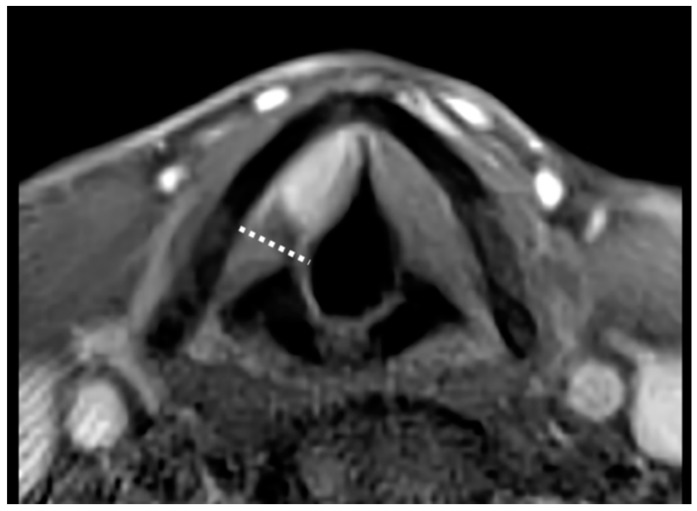
Contrast-enhanced T1-weighted magnetic resonance (MR) sequence showing an endoscopically cT2 glottic tumor (with impaired vocal cord mobility but no arytenoid fixation) turned out to be radiologically cT3 for anterior paraglottic space (PGS) involvement and subsequently confirmed as pT3 at histopathological examination after open partial horizontal laryngectomy (OPHL) Type IIa. Note the abovementioned plane passing through the arytenoid vocal process perpendicular to the ipsilateral thyroid lamina (dotted line), dividing the PGS into anterior and posterior compartments.

**Figure 2:**
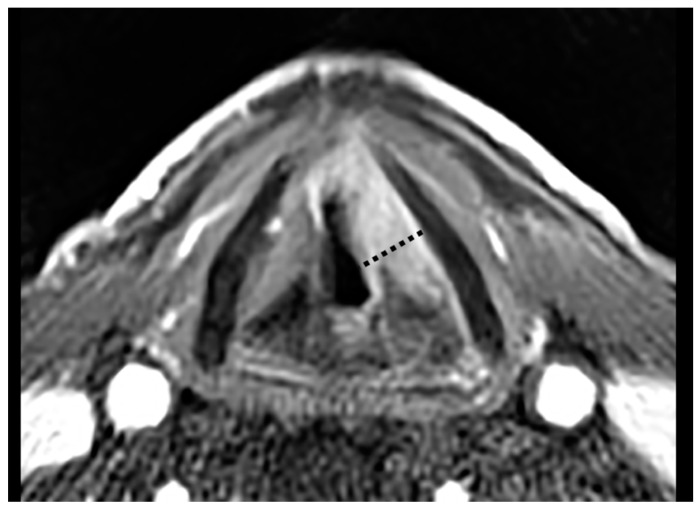
Contrast-enhanced T1-weighted MR sequence showing an endoscopically cT3 glottic cancer (with arytenoid fixation), turned out to be radiologically cT3 and histopathologically pT3 for both PGS involvement and marginal thyroid cartilage infiltration after an OPHL Type IIa + ARY. In this case, the whole PGS was involved but the neoplastic posterior transgression of the abovementioned plane (dotted line) prompted us to include it in the posterior tumor category.

Twenty patients (23.5%) were treated by OPHL Type IIa, 42 (49.4%) by OPHL Type IIb, 13 (15.3%) by OPHL Type IIIa, and 10 (11.7%) by OPHL Type IIIb. All patients underwent central compartment neck dissection (ND). Prophylactic selective (levels II–IV) ND was performed in cases of supraglottic, subglottic, or extra-laryngeal extensions, while therapeutic selective (levels II–V) ND was indicated for preoperative evidence of nodal metastases. Tumor location was retrospectively categorized as “anterior” (Figure 1) or “posterior” (Figure 2) according to its position within the PGS in relation to a frontal plane passing through the arytenoid vocal process and perpendicular to the ipsilateral thyroid lamina [21]. Tumors significantly transgressing in the posterior direction on the virtual plane were defined “posterior”. In cases of lesions equally involving the anterior and posterior compartments, the worst pattern (i.e., the “posterior” one) was considered the most influencing from a prognostic point of view (Figure 2). Classification was based on the analysis of pre- and intraoperative laryngoscopy, radiological examination [computed tomography (CT) and/or magnetic resonance (MR)], surgical reports, and definitive histopathology. Clinical, radiological, and intraoperative data were used to guide histopathologic evaluation in order to provide a reliable definition of the real tumor extension focusing on the PGS. In fact, close cooperation between the surgeon and surgical pathologist was considered an essential prerequisite to adequately define involved and uninvolved structures in such a complex anatomical compartment.

In consideration of the confounding influence of inflammation on MR, patients receiving this kind of evaluation were assessed according to a standardized protocol, previously described in another paper [24]. In this view, MR proved to be extremely accurate in predicting posterior PGS involvement.

Clinical follow-ups were conducted via videolaryngoscopy every 2 months for the first 2 years after surgery, every 3 months for the third year, every 4 and 6 months for the fourth and fifth years, respectively, then continuing with yearly controls. Radiological follow-ups included CT or MR every 6 months and an annual chest CT for the first 2 years, and annual MR/CT and chest CT until the fifth year after surgery. Neck ultrasound was performed regularly every 6 months for the first 3 years. Mean follow-up time was 55.1 months (median, 47; range, 6–148).

### Statistical Analysis

The following endpoints were considered: OS, defined as the time between the date of surgery and date of death/last visit; DSS, defined as the time between the date of surgery and date of cancer related death/last visit; recurrence-free survival (RFS), defined as the time between the date of surgery and date of recurrence/last visit; and laryngectomy-free survival (LFS), defined as the time between the date of surgery and date of total laryngectomy/death/last visit.

The influence of the anterior vs. posterior tumor location on the prognosis was estimated through the computation of OS, DSS, RFS, and LFS curves using the Kaplan–Meier method and comparison by the Log-rank test for dichotomic variables. The same method was applied for other relevant variables: various patient-related factors, T and N categories, perineural (PNI) and lympho-vascular invasion (LVI), and resection margins. Multivariate analysis was performed using Cox proportional hazard models and expressed as hazard ratio (HR) with 95% confidence intervals (CI). GraphPad Prism Version 6.0 (San Diego, CA, USA) and IBM SPSS Statistics version 24.0 were used for the statistical analyses. For all tests, a two-tailed *p* value < 0.05 was considered as significant.

## 3. Results

At histopathology, 67 (78.8%) tumors were classified as pT3 and 18 (21.2%) as pT4a. Thirty-seven (43.5%) were classified as anterior and 48 (56.5%) as posterior. In detail, 26 (30.6%) were categorized as anterior T3, 41 (48.2%) as posterior T3, 11 (13%) as anterior T4a, and 7 (8.2%) as posterior T4a. Two (2.3%), 52 (61.2%), and 31 (36.5%) tumors were, respectively, described as well-, moderately-, and poorly-differentiated SCCs. Eighteen (21.2%) patients had nodal metastases (6 pN1, 5 pN2a, 3 pN2b, 2 pN2c, and 2 pN3b). In particular, 5 pN1 and 2 pN2b patients according to the 7th Edition of the TNM classification system were upstaged to pN2a and pN3b, respectively, following the 8th Edition. PNI was found in 30 (35.3%) lesions and LVI in 22 (25.9%). Table 1 summarizes the main characteristics of patients and tumors.

Complete tumor removal within clear surgical margins (R0) was accomplished in 77 (90.1%) patients, among these, 12 (14.1%) had close resection margins (<5 mm). In the remaining cases, microscopic infiltration of surgical margins (R1) was encountered. Both arytenoids were preserved in 31 anterior (22 anterior T3, 9 anterior T4) and 4 posterior (all posterior T3) tumors, respectively. Conversely, the resection of one arytenoid or crico-arytenoid unit was performed in 6 anterior (4 anterior T3, 2 anterior T4) and 44 posterior tumors (37 posterior T3, 7 posterior T4), respectively. Surgery was followed by adjuvant treatments in 18 (21.2%) patients, 13 (15.3%) received RT and 5 (5.8%) received CRT. In detail, 11 (12.9%) patients underwent adjuvant therapy for nodal metastasis with extranodal extension and/or positive-close margins of resection, 7 (8.2%) for the combination of high stage with positive PNI/LVI.

Overall, 24 (28.2%) patients developed postoperative complications: 10 (11.8%) had a dehiscence of the pexy, 11 (12.9%) required surgical revision for postoperative bleeding, and 4 (4.7%) developed salivary fistula. Other less frequent complications were chylous leak and pharyngeal pouch, each found in one patient (1.2%). Two (2.4%) patients died during the perioperative (30 days) period, 1 from massive hemorrhage and 1 from sepsis.

Four patients had to maintain the tracheostomy due to airway stenosis (3 posterior tumors and 1 anterior tumor). Furthermore, 3 died before decannulation (all posterior tumors). Four (4.7%, all posterior tumors) patients were treated by TL for functional reasons due to major aspiration, despite intensive swallowing rehabilitation; of these, 3 were tracheotomy-dependent due to insufficient airway patency. During follow-up, 30 (35.3%) patients had tumor relapse: 13 (15.3%) local, 8 (9.4%) regional, and 9 (10.6%) loco-regional. The number of loco-regional recurrences for each subcategory was distributed as follows: 5 (19.2%) patients in anterior T3 lesions, 17 (41.5%) among posterior T3, 4 (36.4%) among anterior T4a, and 4 (57.1%) in posterior T4a. Fourteen patients (16.5%) developed pulmonary metastasis. Among the 30 patients who developed loco-regional relapse, 20 (23.5%) underwent surgery (TL and/or ND) and 4 (4.7%) underwent non-surgical treatments. In the latter group, one patient underwent RT for isolated nodal recurrence, while the remaining 3 (3.5%) underwent CRT. Six (7.1%) patients developed untreatable relapse and were managed only by the best supportive care.

At the last follow-up (February 2018), 54 (63.5%) patients were alive without evidence of disease, 18 (21.2%) died of the disease, 8 (9.4%) died due to other causes, and 5 (5.9%) were alive with the disease.

**Figure 3:**
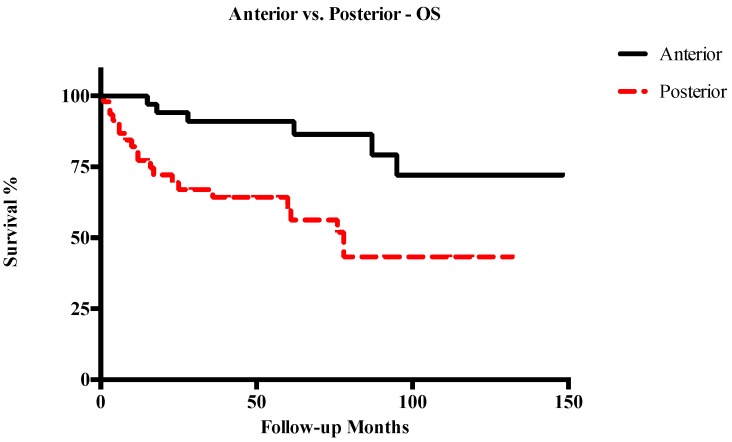
Kaplan–Meier curves representing overall survival (OS) of anterior vs. posterior T3–T4a glottic tumors.

**Figure 4:**
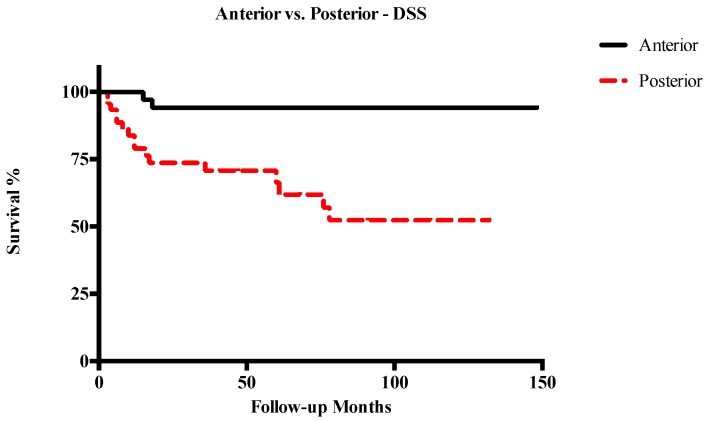
Kaplan–Meier curves representing disease-specific survival (DSS) of anterior vs. posterior T3–T4a glottic tumors.

**Figure 5:**
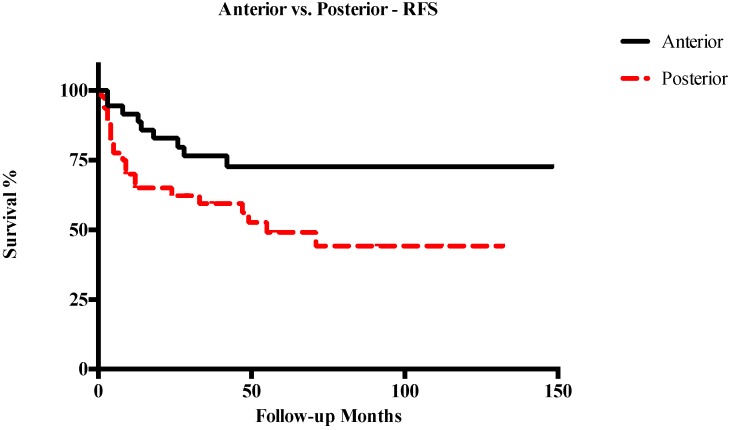
Kaplan–Meier curves representing recurrence-free survival (RFS) of anterior vs. posterior T3–T4a glottic tumors.

**Figure 6:**
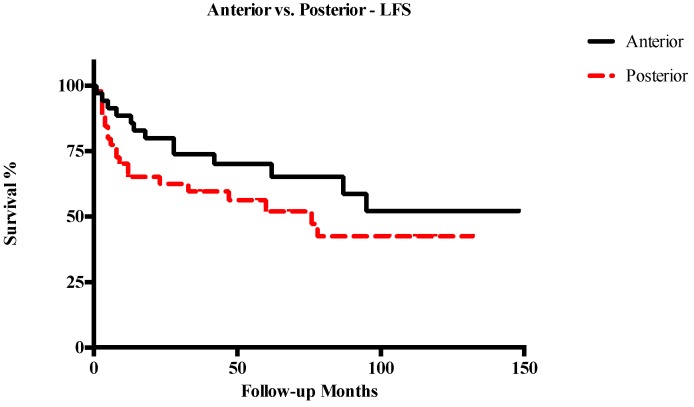
Kaplan–Meier curves representing laryngectomy-free survival (LFS) of anterior vs. posterior T3–T4a glottic tumors.

Five-year OS, DSS, RFS, and LFS in patients with anterior T3–T4a LC were 91%, 94.1%, 72.6%, and 70.2%, respectively. Conversely, in cases of posterior T3–T4a lesions, 5-year OS, DSS, RFS, and LFS were 60.3%, 66.3%, 49.1%, and 52%, respectively. Other survival outcomes are reported in detail in Table 2. In general, posterior tumors had significantly lower OS (*p* = 0.003), DSS (*p* = 0.001), and RFS (*p* = 0.003), while differences in LFS were not statistically significant (Figure 3, Figure 4, Figure 5 and Figure 6). The comparison between different T categories, tumor compartmentalization, and other prognostic factors in univariate analysis are summarized in Table 3.

Multivariate analysis confirmed the findings of univariate analysis. In particular, tumor extension was a significant prognosticator in OS, DSS, and RFS. Similarly, we observed a lack of prognostic impact of the pT category (Table 4). Considering all prognostic factors analyzed, age showed a significant negative impact on prognosis, PNI was confirmed to significantly influence survival in both uni- and multivariate analyses, while N status significantly impacted on RFS only in the latter (Table 4).

## 4. Discussion

The aim of our retrospective study was to evaluate the prognostic impact of tumor location within the PGS (anterior vs. posterior) in advanced LCs treated by OPHLs. Tumors were subdivided according to their compartmentalization in relation to a frontal plane passing through the vocal process of the arytenoid, perpendicular to the thyroid lamina. Patients with tumors macroscopically transgressing this theoretical boundary in the posterior direction had worse clinical outcomes in terms of survival and loco-regional control. This was demonstrated to be an independent prognosticator, it had no correlation with any other prognostic factors and retained its statistical significance even in the multivariate analysis.

In this view, our data support the appropriate indication of OPHLs as laryngeal preservation techniques in the treatment of anteriorly located T3–T4a laryngeal SCCs but raise concerns about the adequacy of such an approach in more posterior neoplasms. In particular, survival analysis showed that posterior T3 lesions had similar outcomes to T4a cancers. For this reason, when OPHLs are the treatment of choice, these tumors should be considered as comparable with T4a tumors in terms of survival outcomes. Furthermore, even anterior T4a lesions demonstrated satisfying results in terms of OS and DSS (both 88.9%) but retained a higher risk of relapse, requiring TL (RFS, 51.1% and LFS, 52.5%). Though different authors [25,26,27] reported the absolute value of the T category as a significant prognostic factor, our data suggest that in locally advanced lesions involving the PGS, a posterior extension through the described plane has a superior prognostic role when compared to the T category itself—at least for what concerns patients and tumors amenable to OPHL. This was first shown by Succo et al. [21], who reported better survival in anterior tumors and showed comparable results between anterior T4a and posterior T3 lesions in terms of OS, DSS, and RFS. However, the prominent prognostic role of anterior vs. posterior laryngeal compartmentalization had yet to be confirmed by an independent series.

The correlation between posterior extension and unfavorable survival outcomes can be mainly explained by the close relationship of posterior PGS with a number of neuro-vascular structures (branches of the superior and inferior laryngeal artery and veins, related lymphatic vessels, and branches of the inferior laryngeal nerve) which can favor a cranio-caudal pathway of spread [28,29,30]. Furthermore, these soft tissues may act as avenues through which the tumor can reach the crico-arytenoid joint and its related muscles, spreading rapidly outside the laryngeal framework into the piriform fossa, esophageal-tracheal groove, and neck [31,32].

Moving to other prognostic factors, age (analyzed as a continuous variable) negatively impacted oncologic outcomes. Naturally, aging can influence OS due to the higher probability of dying from other causes. However, the same prognosticator showed a significant negative correlation with DSS and RFS, raising the question of the role of immune-aging in controlling neoplastic disease. Recently, Yang et al. [33] calculated the importance of age in influencing the prognosis of head and neck cancers as a whole on the SEER database, reporting an especially high impact in laryngeal tumors (16.5% at the Gini index).

The presence of PNI was confirmed to have a significant negative impact in both uni- and multivariate analyses. This finding was previously reported by other authors [34,35,36,37]. In particular, Chirila et al. analyzed a cohort of 256 patients treated for laryngeal and hypopharyngeal cancers and reported the presence of PNI in 35.6% of cases—which corresponds well with the rate observed herein—with a significant impact of minor nerve PNI on DSS and RFS [34].

While the prognostic relevance of N status in LC (as in every head and neck tumor) is usually quite evident, in the present series it appeared to influence only RFS in multivariate analysis. Again, this may reflect a selection bias due to the inclusion of a high proportion of patients with no or just small-volume lymphadenopathies in the present cohort.

Overall, our survival outcomes and surgical complication rates are in line with those reported in the literature, confirming the feasibility, efficacy, and reproducibility of OPHLs [3,20,25,38,39,40]. In a systematic review on OPHL for LC including all T categories, Thomas et al. [41] reported a 2-year OS and DSS of 79.7% (*N* = 3964) and 84.8% (*N* = 2344), respectively.

The main drawbacks of our study can be found in the retrospective nature of the analysis, conducted on a relatively limited cohort of patients, in which 43.5% of cases had been previously treated for LC by TLM. Moreover, functional outcomes were not detailed as they have already been extensively described elsewhere and are out of the scope of the present analysis [42,43,44]. Finally, our series included fit and motivated patients, intentionally selected to be treated by a specific therapeutic option such as OPHL, presenting relatively limited tumor and nodal burden. Therefore, further studies are required to confirm the importance of laryngeal anterior vs. posterior compartmentalization in patients to be managed by other surgical (such as TL, TLM, or transoral robotic surgery) and non-surgical treatments. Indeed, adjunctive data may provide sufficient validation to help in the refinement of the current TNM classification for LC, by upgrading the impact of posterior PGS involvement. Moreover, multicentric prospective analyses are desirable to clarify the heterogeneity of T3 and T4a LCs, define effective prognostic factors, and reach a consensus on the ideal treatment strategy to be specifically tailored based on patient- and tumor-related features.

## 5. Conclusions

Treatment of T3 and T4a LCs still represent a challenge for the head and neck surgeons, radiation, and medical oncologists. The literature is rich of meta-analyses and original papers comparing different surgical and non-surgical strategies. Nonetheless, due to the retrospective nature of most studies—the large heterogeneity of lesions included in T3–T4a categories and impossibility to completely match all cancer- and patient-related features—sound criteria to select the most appropriate treatment for a specific tumor are still lacking. More studies are required to identify factors that can lead to favor a surgical vs. non-surgical approach. In the absence of a biological marker or genetic signature thus far that can guide treatment selection, the identification of a reproducible clinical parameter able to separate “good” from “poor” OPHL candidates in relation to the extent of the primary within the PGS, should be regarded as a paramount element for the everyday decision-making process. Further studies are needed to demonstrate the impact of tumor location in different surgical approaches as well as in non-surgical protocols.

## Figures and Tables

**Table 1:** Principal features of patients and tumors in the anterior and posterior compartments.

Category	Anterior Location	%	Posterior Location	%
Numbers of patients	37		48	
Mean Age	60.7		60.7	
T3	26	70.3	41	85.4
T4	11	29.7	7	14.6
N+	7	18.9	11	22.9
PNI+	12	32.4	18	37.5
LVI+	10	27	12	25
Positive resection margins	4	10.8	4	8.3

**Table 2:** Five-year survival outcomes of the entire study population.

Category	OS %	DSS %	RFS %	LFS %
Overall	74.1	79.1	59.4	59.9
Anterior	91	94.1	72.6	70.2
Posterior	60.3	66.3	49.1	52
T3	74.1	80.5	63.4	63.8
T4	71.8	71.8	43	43.1
Anterior T3	91.6	96	79.6	75.2
Posterior T3	61.7	69	52.4	56
Anterior T4	88.9	88.9	51.1	52.5
Posterior T4	50	50	34.3	34.3

**Table 3:** Univariate analysis of prognostic factors (*p* value).

Prognostic Factors (p)	OS	DSS	RFS	LFS
Anterior vs Posterior	0.003 *	0.001 *	0.003 *	0.167
T3 vs T4	0.802	0.462	0.446	0.426
Anterior T3 vs. Posterior T3	0.017 *	0.004 *	0.027 *	0.108
Anterior T4 vs. Posterior T4	0.107	0.044 *	0.573	0.867
Anterior T3 vs. Anterior T4	0.672	0.423	0.219	0.248
Posterior T3 vs. Posterior T4	0.503	0.302	0.679	0.695
Posterior T3 vs. Anterior T4	0.189	0.167	0.614	0.934
Age	0.002 *	0.008 *	0.027 *	0.113
N0 vs N+	0.248	0.142	0.135	0.319
PNI	0.035 *	0.034 *	0.019 *	0.050 *
LVI	0.592	0.837	0.685	0.645
Positive resection margins	0.586	0.564	0.623	0.558

* Statistically significant *p* values (<0.05). The same as below.

**Table 4:** Multivariate analysis of age, tumor extension, T category, perineural (PNI), and nodal status on OS, DSS, RFS, and LFS.

Prognostic Factors	*p*-Value	HR	95% CI	
Multivariate OS				
Anterior vs. Posterior	0.02 *	3.10	1.23	7.80
T3 vs. T4	0.72	1.19	0.46	3.09
Age	0.001 *	1.10	1.04	1.17
N0 vs. N+	0.15	2.32	0.74	7.33
PNI	0.007 *	3.73	1.44	9.66
Multivariate DSS				
Anterior vs. Posterior	0.004 *	9.10	2.00	41.43
T3 vs. T4	0.32	1.74	0.58	5.19
Age	0.01 *	1.10	1.02	1.17
N0 vs. N+	0.08	3.54	0.87	14.40
PNI	0.02 *	3.83	1.23	11.98
Multivariate RFS				
Anterior vs. Posterior	0.03 *	2.51	1.12	5.64
T3 vs. T4	0.31	1.58	0.66	3.77
Age	0.01 *	1.07	1.02	1.13
N0 vs. N+	0.02 *	3.15	1.22	8.14
PNI	<0.001 *	5.75	2.31	14.32
Multivariate LFS				
Anterior vs. Posterior	0.91	0.95	0.38	2.35
T3 vs. T4	0.41	1.51	0.57	4.00
Age	0.08	1.05	0.99	1.11
N0 vs. N+	0.59	1.42	0.40	4.98
PNI	0.002 *	5.26	1.86	14.88
